# Vulnerability and Complexity: Wartime Experiences of Arab Women During the Perinatal Period

**DOI:** 10.3390/ijerph22040588

**Published:** 2025-04-09

**Authors:** Maram Awad-Yasin, Lia Ring, Elad Mijalevich-Soker, Orit Taubman – Ben-Ari

**Affiliations:** 1 The Louis and Gabi Weisfeld School of Social Work, Bar-Ilan University, Ramat Gan 52900, Israel; yaseenm1@biu.ac.il (M.A.-Y.); liaring@biu.ac.il (L.R.); 2 Department of Psychology, Bar-Ilan University, Ramat Gan 52900, Israel; elad.mijalevich@biu.ac.il

**Keywords:** perinatal period, women, war, Arab, minority

## Abstract

Pregnancy and transition to motherhood are significant experiences accompanied by manifold changes, particularly during times of crisis, such as exposure to traumatic events, which involve further levels of complexity and vulnerability. This is especially true for Arab women in Israel, considering the interactions between their physical, mental, and social environments, and their impact on health during pregnancy and childcare in wartime. This study sought to examine the experience of Arab women in Israel, who experienced intersectional marginalization as women in a traditional patriarchal society who belong to a minority group, during the perinatal period, following the events of 7 October 2023, and the subsequent Israel–Hamas war. Semi-structured in-depth interviews were conducted with ten participants in different stages of pregnancy and young mothers to infants up to a year old. The thematic analysis revealed four main themes: *The impact of the war on intrapersonal identity*; *The impact within the couple’s relationship identity*; *The impact on family identity*; *Socio-political identity*. Shining a spotlight on the experiences of Arab women in Israel and their daily functioning in the current complex reality reveals unique challenges, encompassing profound feelings of fear, anxiety, and imposed silence. Deepening the understanding of marginalized women’s experiences can help policymakers in the field of women’s health to design tailored adaptations in health policies for Arab women from minority groups, navigating the complexities of transitioning to motherhood during periods of social instability and wartime challenges.

## 1. Introduction

Pregnancy and the transition to motherhood are significant experiences that are accompanied by a wide range of physical, emotional, and social changes for women. These changes involve happiness and expectations, alongside stress and anxiety [1]. The perinatal period of pregnancy and early motherhood demands significant adaptation for women, including adjusting to changes in identity and couple relationships. Social and family support systems play a pivotal role in assisting women to navigate their path and to ensure mothers’ and their future children’s well-being [2].

During times of crisis and war, the experiences of pregnancy and new motherhood take on additional layers of complexity and vulnerability [3], since, in these turbulent times, future mothers must cope with increased challenges that go beyond the typical concerns of parenthood. Extreme events, such as war, can dramatically disrupt women’s access to healthcare, making it challenging to obtain prenatal care, safely give birth, or receive postpartum care [4]. Such circumstances can harm women’s mental health, thus increasing the risk of experiencing stress and developing postpartum depression [5] and also harming their ability to care for their children and their relationships with the children [6]. This may also result from damage to the social fabric, including family and community support [7]. Hence, it is essential for policymakers in the field of mental health to take responsibility for raising awareness among decision makers about the mental health consequences of armed conflicts. This responsibility includes addressing the needs of individuals and communities affected by war, providing support, and fostering resilience [8].

The consequences of crises for women during the perinatal period are even more prominent for minority populations, such as Arab women in Israel, who already face multiple barriers to medical care in routine times [9], and during crises such as the COVID-19 pandemic [10,11]. Arab mothers in Israel face significant barriers to accessing healthcare, including reduced economic resources and limited use of both formal and informal support services [12]. These barriers are shaped by cultural stigma around mental health within the Arab community and a lack of culturally tailored services. Their vulnerability is also due to belonging to a traditional patriarchal society [13,14], and is exacerbated as the geo-political tensions add to feelings of insecurity and instability that impact these women’s daily lives [15] and their functioning as new mothers [5]. In this context, Arab women face a three-fold vulnerability, related to their intersection in multiple marginal positions [16,17]: as women in a patriarchal society, as members of a minority group, and as women during the perinatal period.

On 7 October 2023, the Hamas terrorist organization launched a deadly attack near Israel’s Gaza border, killing, injuring, and abducting citizens. This initiated a war between Israel and Hamas, while residents of Israel endured daily missile attacks, had to evacuate their homes, experiencing loss and bereavement, all of which has deeply impacted Israeli families, leading to heightened distress [18].

This study seeks to elucidate the impact of such turbulent times on Israeli Arab women’s experiences of pregnancy and infant care. The research question guiding this inquiry is as follows: how do Israeli Arab women in the perinatal period perceive and experience the events and their own role as childcare providers?

## 2. Method

### 2.1. Participants and Procedure

After this study was approved by the Institutional Review Board, participants were recruited through convenience sampling from birth preparation classes, social media, and referral from interviewed women. This study was conducted in Arabic by the first author, a community social worker and a researcher from the Arab society in Israel. Data were collected through semi-structured in-depth interviews conducted via Zoom.

The interviews lasted between 40 and 90 min and were conducted 2 weeks to 1 month after the terror attacks on 7 October 2023, and the Israel–Hamas war that erupted immediately afterward. Participants were informed about the research topic and goals, gave informed consent to participate and to be recorded, and were promised confidentiality regarding identifying details.

Data were recorded, transcribed verbatim in Arabic, and subsequently translated into Hebrew by the first author, who is fluent in both languages. Identifying information was removed during transcription, and files were stored on a password-protected encrypted device.

This study included ten women aged 25–33 (*M* = 29, *SD* = 2.74), all Israeli-born, married, and members of the Arab minority in Israel. The sample consisted of eight Muslim women, one Christian woman, and one Druze woman. Five participants were in their first pregnancy, three were pregnant and had another young child, and two were mothers of infants under one year old. Participants’ professions included nursing, teaching, law, and other roles, while one participant did not work (for more information, see Table 1). Recruitment was concluded when thematic saturation was reached, as no new themes or insights emerged from additional interviews.

### 2.2. Instruments

The interview was based on key questions to encourage the development of descriptions by the participants related to their worldviews and their lives, allowing for additional questions according to the interviewee’s reflections. First, participants were asked about background information (age, marital status, profession, number of children, and/or pregnancy period). Second, they were asked about their experience of pregnancy and/or motherhood in general. Then, the participants were asked to describe their experiences when they first heard about the events of 7 October 2023, and how these events impacted their lives since then. The interview concluded with a discussion about the future, giving participants the opportunity to share any additional topics or concerns that were not addressed during the interview.

The interview protocol was developed by the research team for clarity and cultural appropriateness. While participants did not contribute directly to the formulation of the questions, they were given space during the interviews to guide the conversation and introduce topics of relevance to them, reflecting a semi-participatory approach.

### 2.3. Data Analysis

**Figure 1:**
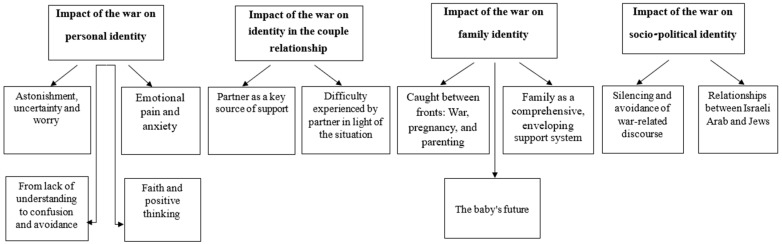
Map of research themes.

A thematic analysis was conducted by the authors to identify recurring issues (themes) within the data, following the approach outlined by [19]. This method adopts a “bottom-up” approach, linking the identified themes directly to the dataset. After reading and re-reading the transcripts, a data infrastructure was established, incorporating detailed records of initial ideas and observations. Subsequently, preliminary codes were generated, encompassing the entire dataset, with relevant quotes linked to each code. In the next stage, these codes were amalgamated to form potential themes, and the researchers developed a comprehensive thematic map (Figure 1) to visualize the relationships between the themes.

Trustworthiness was established by adhering to the phases of qualitative research as outlined by Braun and Clarke, ensuring consistency and reliability in the findings derived from this methodological framework [20]. Multiple strategies were employed, and an audit trail documented all stages of the research process, including coding decisions and thematic development.

The research team included both Jewish and Arab researchers, men and women, from the fields of psychology and social work. The first author, who conducted all the interviews, is a community social worker and a researcher from the Muslem–Arab society in Israel. Her shared cultural and linguistic background with the participants facilitated rapport and deepened the interview process. At the same time, the researcher remained aware of her own positionality, including her professional training, personal experiences of the national trauma, and emotional proximity to the participants’ narratives. The other team members consisted of two research psychologists, whose expertise includes transition to parenthood, parenting, and multiculturalism, and a clinical social worker, who is also a researcher, specializing in trauma, who works therapeutically with couples and families.

Triangulation was conducted through collaborative analysis involving all authors, who discussed and refined themes iteratively. Emerging themes were compared across interviews to ensure consistency and resonance with participants’ narratives. The diversity of the research team—including both Jewish and Arab researchers from the fields of psychology and social work, men and women—contributed to a richer and more nuanced analysis. Discussions within this multidisciplinary and multicultural team helped shape the interpretation of the findings, particularly in relation to issues of cultural sensitivity and context. Team consultation assisted in acknowledging potential biases, enhancing analytical transparency. The final themes and subthemes were refined, defined, and presented in a narrative framework that highlighted their relevance to this study’s context. For the purposes of this research, the analysis focused on Arab women in Israel, addressing their experiences of national trauma, their vulnerabilities, and the complexities of being women from a minority group navigating the transition to motherhood and pregnancy.

## 3. Results

Thematic analysis of the interviews yielded four themes related to the participants’ experiences, feelings, thoughts, and perceptions during the war: (1) The impact of the war on personal identity, relating to immediate emotional reactions to the emerging reality, comprising four subthemes: astonishment, uncertainty, and worry; emotional pain and anxiety; lack of understanding, confusion, and avoidance; and faith and positive thinking. (2) The impact of the war on the couple relationship identities, consisting of two subthemes: partners as a central source of support; and partners’ difficulties in light of the situation. (3) The impact of the war on family identity, comprising three subthemes: caught between fronts: war, pregnancy, and parenting; family as a comprehensive enveloping support system; and the baby’s future. (4) Socio-political identity, consisting of two subthemes: silencing and avoidance of war-related discourse and the relationships between Israeli Arab and Jews. A schematic description of the themes and subthemes appears in Figure 1.

### 3.1. Theme 1: Impact of the War on Personal Identity

Participants reflected on the impact of the war on their personal identities, such as concerns about the situation and loved ones, negative feelings in light of what was happening, choices to avoid exposure to what was happening, and positive thinking as a mechanism for improving emotional resilience.

#### 3.1.1. Subtheme 1.1. Astonishment, Uncertainty, and Worry

Participants expressed shock and astonishment, feeling their familiar world had been shaken. Worry and uncertainty were prevalent. The descriptions of 7 October 2023 were dramatic, with phrases like “the door to hell”, “world war”, and “panic”, reflecting a sense that this was an unprecedented event, unlike anything they had experienced before.

“Perhaps this is a situation that has opened the door to hell for us, we are entering the unknown and there is doubt about what we will do and what will happen in future… The country has no answers at all to where all this is going”.

(Interviewee 1)

“You put the worst-case scenario in front of you. Like, is there a chance that a major war will break out? Your thoughts go to the farthest places…Where will this lead us? It was clear that [the event] this time is different [from events] that preceded it. Therefore, we didn’t know when it would end, where it would lead, and how the situation would look”.

(Interviewee 5)

“I am more afraid about what will happen… They are talking everywhere saying this is the beginning of a world war. It could be or not, no one knows, and if it starts—what will happen to us?”.

(Interviewee 3)

“I felt worried, about how to continue living, what will happen to the girl [daughter], where will she go?”.

(Interviewee 4)

“This situation is scary and worrying… war, when you hear about it for the first time, there is great fear”.

(Interviewee 9)

#### 3.1.2. Subtheme 1.2. Emotional Pain and Anxiety

The participants expressed pain regarding what was happening and recounted their thoughts about the situation in the country, and the lack of stability led them to deep feelings of nervousness, sensitivity, and a feeling of living in a “nightmare”. These feelings led them to envision extensive negative outcomes, further heightening their anxiety levels.

“…there’s also pain for every person who has died without fighting, to give legitimacy to killing people is not human… there was pain from the consequences of the subject”.

(Interviewee 1)

“It wasn’t easy, and I also, in the initial period… in the first 3–4 days of this period, I had a very hard time… an unpleasant feeling, I felt anger and nervousness”.

(Interviewee 3)

“I was scared, I was anxious, I felt it wasn’t like always, this time I felt it was completely different…”.

(Interviewee 8)

“There was stress and fear at the same time. Stress because nothing is known because we are going to an unknown place”.

(Interviewee 5)

#### 3.1.3. Subtheme 1.3. From Lack of Understanding to Confusion and Avoidance

Initially, the women felt confused and unsure of what had happened. Even after watching the news and social media, they struggled to believe and absorb the events, leading to a sense of denial and bewilderment. Their descriptions, often using imagery of sleep and dreaming, reflected a feeling that the situation was unreal.

“I felt that I was in a dream”.

(Interviewee 2)

“As if a person was astonished and trying to understand what is happening and doesn’t understand, that is, fell asleep and woke up to the Holocaust. It was very difficult”.

(Interviewee 3)

“When I read [the messages] I didn’t understand exactly what it was because of the size of the event. I didn’t understand whether the events described were true or were a prank. My husband came and explained it to me. I remember that I couldn’t absorb what he said, I managed to fully digest what happened only after one, two, three days… I managed to understand what’s happening and to understand the situation the country is in”.

(Interviewee 7)

The interviews revealed that participants consciously chose to stop watching the news, with some even deleting social media accounts to avoid updates. Initially, the constant exposure heightened their anxiety, so disconnecting and avoiding media became a coping strategy. The recurring theme was “deletion”, reflecting their desire to avoid violent and distressing content.

“It was very difficult… I even felt that I had no appetite and lost weight… I was very affected… I would watch the news or read the news and then I decided to stop. Even today I don’t see anything on the news…”.

(Interviewee 10)

“I followed everything during the first three days until I decided to stop watching the news. After that, I decided to only follow updates on Facebook or social media. Recently, I decided to give up on social media too and not to know anything”.

(Interviewee 3)

“The best step I took was deleting Instagram, to let myself breathe, and not to bring in negative energy, not into the home and not to my daughter, because I’m one of those people who those videos impact, and those stories that a mother lost her child and baby impact me a lot. It’s very hard for me and therefore, that’s it, I deleted it”.

(Interviewee 4)

“Currently I don’t watch the news. I am immediately hurt when I see the news, even my husband opens his telephone and watches the news or television at my uncle’s house and also at my parents’ house—the same thing and loudly, everyone follows the news throughout the day. It disturbs me emotionally, I am exhausted, so I asked them to change the channel—and if it’s on the phone—to skip over it”.

(Interviewee 6)

“The more I watched the news, I would cry and cry and cry until I set a boundary for myself that I am forbidden to watch the news anymore. Enough…”.

(Interviewee 7)

#### 3.1.4. Subtheme 1.4. Faith and Positive Thinking

Participants shared aspects of emotional resilience and positive ways of coping during this stressful period, such as turning to religion and relying on faith to receive consolation and security, along with positive and reinforcing self-talk that helped them regulate the anxiety they felt:

“Nothing else will help us, other than strengthening ourselves with positive talk. To sit and say that things won’t work out—we will harm ourselves emotionally, and that will go against us. We need to strengthen ourselves with positive talk and in the end, everything will pass”.

(Interviewee 6)

“I am usually a positive person. I never perceived myself as someone who thinks negatively. Many times, just the positive thoughts [help]. If something [bad] is going to happen, then it will happen, there is no way to prevent it. These are things that helped. Also sport”.

(Interviewee 2)

“I would read the Quran a lot, that’s what helped me”.

(Interviewee 4)

“I started to pray and told myself that everything is written by our God… Everything is in God’s hands… He gave me the faith and thus the fear diminished and calmed the soul”.

(Interviewee 8)

The quotes reveal that the women relied on their faith as a primary coping strategy, which fostered emotional resilience. They also used positive affirmations and thoughts to help manage the situation. These women demonstrated the ability to face the challenges of the war through their inner strength, optimism, and hope for a better future.

### 3.2. Theme 2: Impact of the War on Identity in the Couple’s Relationship

Participants described in detail the impact of the events on their couple relationships. This theme comprised two subthemes: partner as a key source of support; and the need to support the partner in light of the situation.

#### 3.2.1. Subtheme 2.1. Partner as a Key Source of Support

Participants mentioned their spouse as an important source of support in these troubling times. They felt they could lean on them and trust their helping presence and protective shield.

“Thank God, I have my partner [said with a smile], he supports me at every moment, I always share everything with him. It helps me”.

(Interviewee 2)

“The first one to support me is my husband. He is the first person who is with me at home, it’s also my personal opinion that if the husband is supportive—then it’s enough. My husband helps me with housework and takes me out of the house—even if it’s half an hour… he shares other things with me, prepares food and everything”.

(Interviewee 3)

“The one who helps me the most, and the most important, is my partner. He helps me in the house and with the girls, and [we] also talk to each other, which lowers [the pressure] on me a bit. We are both suffering from the same complexity”.

(Interviewee 5)

“What most helps me is my husband thank God. He helps me, supports me, and accepts me… and even appreciates me more when he sees how much the woman suffers during pregnancy. [He asks] what I am lacking, what I want… he is always considerate in these matters, especially the appointments with the doctors. At first, he would make the appointments”.

(Interviewee 6)

“My husband was with me in everything. He takes her [the baby] from me at night to let me sleep from 22:00 until the morning, and that is daily and not one day yes one day no. I am telling you honestly, every day—he would take her”.

(Interviewee 4)

From the quotations above, it can be seen that the partners were a meaningful source of support in this emotional situation. Their assistance was expressed both in emotional and instrumental support, i.e., women shared that their partners were considerate of their situations, provided a listening ear, assisted with household tasks, and helped with the infants and other children.

#### 3.2.2. Subtheme 2.2. The Need to Support the Partner in Light of the Situation

Some of the participants shared that they felt that they were an important source of support for their partner, because their partner’s emotional situation was far from good, which mobilized them to be a resource for their partner while they also needed support.

“… He had a whole week where he lay in bed. He didn’t do anything; he didn’t even eat and barely drank some sage tea… I started to keep my distance from him… I started to tell him that I didn’t want to see you in this state… I try to take pressure off him, like payments, or I don’t tell him that I want to buy a certain item of clothing or things that I can live without. I can help him and help myself; the main thing is that this period doesn’t hurt me or the little girl”.

(Interviewee 4)

“I see that he is stressed. Also economically, because he wants to ensure all our needs and our son’s [needs]. This is the most important thing, he doesn’t want to be in a place where he can’t meet our needs, because then he is under stress and pressure. He tries not to show me, but I feel it. When I share with him, I do it calmly, because I don’t want to pressure him more. I always wish him success and try to raise his spirits with positive things”.

(Interviewee 6)

“The people at my husband’s work come from faraway places… then he had to be in their place, and he was away from home for more hours. This increased his stress at home, he felt more responsible and nervous, was busier, and was dominated by feelings of pressure and fear…”.

(Interviewee 9)

These quotes illustrate the challenges the women faced with their partners, who were preoccupied with their concerns, primarily related to income and work amidst the uncertainty and stress caused by the war. At times, the partners were even seen as a burden, leading to conflicts in their relationships. To manage this, some women chose strategies like distancing and avoiding contact to prevent further strain between them.

### 3.3. Theme 3: Impact of the War on Family Identity

The third theme focused on the impact of war on family identity, consisting of three subthemes: caught between fronts: war, pregnancy, and parenting; family as a comprehensive enveloping support system; and the baby’s future.

#### 3.3.1. Subtheme 3.1. Caught Between Fronts: War, Pregnancy, and Parenting

Participants noted the complexity of coping with other children alongside pregnancy and in light of the precarious security situation in the country. The participants’ statements emphasize the increased worry and the fears they experienced in this period. The women shared their ceaseless worrying, and that, in some cases, they were not focused on their pregnancy but on their other children at home, since they had to worry about protected space for them and were occupied with mediating the situation.

“I don’t know how long the war will continue or what will happen when I give birth… At some point, I said to myself that everything was messed up. Just thinking about it is exhausting. In the meantime, I am not functioning with my daughters because my thoughts are constantly at work, and also because I’m tired. I am not managing to focus on the pregnancy because of the situation and because of my daughters… I feel as if I’m torn between several spaces and at the same time I don’t know where I am standing”.

(Interviewee 5)

“…In those days I didn’t even remember I was pregnant; I forgot about it. Sometimes I felt guilty, because I’m also responsible for another soul [the fetus], someone also needs to worry about her. Despite this, I was forced to cope with events that were larger than me… [when] there’s another child with you in the house—it’s much more frightening… even with the return to educational frameworks to routine two weeks ago, my fear grew… the educational frameworks in it aren’t set up and they aren’t prepared for emergencies, so, until they [the local council] take care of things, I have to cope with the question—where should I leave my daughter when I go to work?”.

(Interviewee 2)

“…Usually, a pregnant woman’s hormones change and she is afraid to give birth. Following the events, I don’t ask how I will give birth, but what will happen to my children?… How will I deal with the safety issues? If I want to leave the house and the birth process begins to happen, what will I do?….

(Interviewee 9)

#### 3.3.2. Subtheme 3.2. Family as a Comprehensive Enveloping Support System

Participants described their families of origin and their husbands’ families as supportive (surrounding and enveloping them), whether speaking of physical relief (encouraging them to rest), instrumental (for example, help with cooking), or economic. Moreover, many families were experienced as a significant supportive factor by providing a listening ear and acceptance given the security situation.

“When I’m stressed, I meet my parents, they calm me down, stand by my side and understand me. When they are next to me, I feel support and strength and am not afraid… I get my strength from them”.

(Interviewee 10)

“My mother comes and helps me prepare food and also with the housework, on an emotional level they (the parents) always talk to me and calm me down… One day I was sitting with my husband’s parents in the yard, and we heard the sirens. I felt that everyone was concerned first of all that I would be safe… I felt that I had an escort and I was not alone…”.

(Interviewee 8)

“My husband’s parents live below us in the same building. They supported me a lot, tried to help with tasks that I needed to do at home, and did things for me so that I could rest and not exert myself. My husband and I are from the same town, and my parents live in the same town. They also helped me a lot, they came to my house, checked if I needed anything, invited me to come to their house to change the atmosphere a little sometimes”.

(Interviewee 3)

“On the days when my husband slept outside the home, I was afraid to stay at home alone, at least in the beginning. Therefore, I used to sleep at my parents’ house until the whole period passed. I have an excellent relationship with my mother and with my sister. This makes it easier for me to share with them”.

(Interviewee 2)

#### 3.3.3. Subtheme 3.3. The Baby’s Future

Throughout the interviews, significant experiences of uncertainty about the baby’s future emerged as a central theme. Participants described their efforts to cope with the situation while grappling with constant concerns about their children’s futures—both for those not yet born and those who had recently been born. In other words, the expansion of the family, which in normal times is perceived as a happy event, was, instead, perceived during this threatening period as a source of worry and anxiety.

“I thought to myself—how will my daughter grow up? How will she live? What if she is exposed to hatred? What if someone treats her with racism? How will she cope with this?”.

(Interviewee 7)

“It’s a sense of fear for her… I love her [her daughter] and want her to stay with me, so I am always afraid for her. It’s like, she is mine, born from God, I don’t trust someone else to raise her or to do something for her. There was great fear—and now anything I do—I take her with me: in the shower, in the toilet, to bathe, to cook, and even to sleep. I don’t even want to close my eyes… I didn’t sleep at all for several days so that she would be next to me, and I would protect her”.

(Interviewee 1)

“What scares me the most is what will happen to my children. I want my son to be born into a safe atmosphere, with less conflict between Arabs and Jews… I want him to be born in an atmosphere that isn’t dangerous… That he will live in stability, that I won’t be afraid for him. My son is the most important to me in life, I don’t want him to arrive in stress”.

(Interviewee 6)

The participants expressed fears about the current reality, and felt they were bringing their children into a chaotic and challenging world where coping might be difficult. They emphasized a desire to create a comfortable and safe environment for their children, but the present circumstances made it hard for them to envision how to achieve that.

### 3.4. Theme 4: Impact of the War on Socio-Political Identity

The fourth theme dealt with the impact of the war on socio-political identity, through a focus on two subthemes: silencing and avoidance of war-related discourse; and the relationships between Israeli Arab and Jews. These two subthemes reflected changes for the worse in the social aspects of the participants’ lives.

#### 3.4.1. Subtheme 4.1. Silencing and Avoidance of War-Related Discourse

This subtheme presents the conflict that the participants experienced regarding expressing their position on the situation in Israel. The women claimed that they chose to be silent and not express their positions, partly because of the vagueness of the situation, partly out of fear of the responses of their environment, and partly because any response was likely to be perceived as taking a political stand.

“If someone asks me any question about the war, I will not answer him. I prefer to be distant. The solution is—not to talk about this subject”.

(Interviewee 3)

“How should I answer someone who makes extreme statements in front of me? Or is it preferable not to answer at all? Before [the war], I would answer regularly, not extremely, but I did have the possibility of answering him in a respectful and understanding way. However, this war is different. [During] this war, it’s preferable not to answer at all, it’s preferable not to speak. This war changed the balance [of power]. It is not similar to what was before. Before this, you could imagine that you were permitted to say certain things. Right now, it’s not [like that], you feel that you are forbidden to say one letter about your feelings”.

(Interviewee 7)

“When I am with people, I prefer to speak as little as possible and not to express a position, so that whoever is sitting with me—will feel that each one lives in a different world”.

(Interviewee 4)

From the quotations above, it is evident that there was complexity and difficulty for the participants in expressing a political attitude or an emotional stance regarding the current situation in Israel. The participants consciously chose to avoid expressing their positions due to the complexity of Jewish–Arab relations. This seemed to be a defensive action, designed to protect them from possible hostile reactions and therefore they chose to remain silent.

#### 3.4.2. Subtheme 4.2. The Relationships Between Israeli Arab and Jews

This subtheme dealt with the complexity of Arab–Jewish relations in Israel at this time, especially in the workplace, where encounters were emotionally charged due to tension in relationships that existed before the war and exacerbated due to the stressful situation.

“I work in Jewish educational frameworks, and there it is more difficult. When people hear you speaking Arabic, they immediately [mark you] with a red mark…”.

(Interviewee 2)

“What frightened me at the beginning of the war was that I work in a hospital… there are both Jews and Arabs… I remember that at the start of the war, Jews frequently confronted us verbally…If the situation had continued this way, I would not have been prepared to continue my work, because I did not want to work in a place where I don’t feel comfortable going there”.

(Interviewee 5)

“I hear that the whole issue of relations between Jews and Arabs is especially complicated in a war because Arabs are not able to speak in workplaces. Because if they express their opinions, it will be considered that they think against [their Jewish colleagues], every word is perceived differently and perhaps you will lose your position”.

(Interviewee 1)

“I thought, how will I meet my colleagues, Arab and Jewish? Since I’m not a politician, I was afraid to speak about politics… The following day, I couldn’t decide whether to go to work or not. I work in a hospital, and by evening I decided not to go… I had a fear that they would make faces, or they wouldn’t talk to me, or they would turn on me”.

(Interviewee 3)

“I started to doubt my ability to go to work and to meet people from other nations… What helped me a little was that the Jews who worked with me at the same workplace left [to join] the army for the emergency call-up order. Perhaps that made it easier for me in this situation, or it is possible that the situation became more difficult because I held a certain position without meeting the other side… Specifically, I avoid going to places like this when they are crowded with Jews. This all affected the period of my pregnancy; it has become much more depressing”.

(Interviewee 7)

This subtheme presents the tension between Jews and Arabs, which were present before the events of 7 October 2023 but took on new dimensions in their wake. These led the participants to cope with feelings of oppression, silence, and fear, impacting their functioning in the workplace and harming their sense of capability, something that only increased the tension and fears stemming from the conflictual reality in the country.

## 4. Discussion

Few studies have discussed how Arab mothers cope during the perinatal period in general [17,21], or specifically in wartime. Therefore, the current study, which examined the experiences of pregnant women and new mothers in Arab society in Israel in the wake of the events of 7 October 2023 and the subsequent war, makes an important contribution. The research findings expand the existing knowledge and point to the intersection of personal, couple, familial, and socio-political identities that are intertwined in the experiences of the complex reality of these women’s lives in the shadow of war. These intersections emphasize the vulnerability of Arab women during this sensitive period in the life cycle.

The first theme that arose in the interviews, the impact of the war on personal identity, discussed the emotional responses of the participants to the emerging reality, relating to feelings of shock and uncertainty, fear and anxiety, and confusion and avoidance on the one hand, and relying on faith and positive thinking as coping resources on the other hand. It is evident from the interviews that participants were forced to cope with three fronts during wartime: the front of *vulnerability* stemming from their being pregnant women and/or new mothers, two sensitive periods in the life cycle [22]; the front of *complexity* that they encounter by belonging to a minority group, that is, being Arab women in Israeli society; and the *functional* front, related to the ways they are required, at both the universal human level and the personal level, to manage their daily lives at an individual level, as part of a couple, with their families, and at the social/collegial level.

Although security events and wars impact the entire population, and certainly people during sensitive periods in the life cycle, it is especially important to address the fact that the participants are women from a minority group, which places them at the intersection of marginal positions in society, and these two identities also intersect with the period in which they are obliged to cope with stressful and traumatic events [17]. From the theoretical perspective of the Intersection of Marginal Positions [16], women who belong to minority groups may be excluded or pushed to the margins during routine times. However, the events of 7 October 2023 which were carried out by the operatives of a Muslim terror organization [23], led to a complex situation for the relations between Jewish and Arab citizens of Israel and to an increase in conflictual feelings toward Arabs.

Hence, Arab women in Israel may have experienced three-fold exclusion as they were pushed to the social and familial margins due to being women in a patriarchal Arab society, being part of a minority group in Israeli society, and being in the perinatal period, with all these exclusions being exacerbated during wartime. The situation is even more complex because most of the women interviewed were Muslim, and may have been identified by others, at least to some extent, as siding with the attackers.

The research findings reflect this intersectional identity across personal, couple, familial, and socio-political dimensions. These marginalized positions shape how Arab women perceive their perinatal vulnerability amid the challenges of wartime daily life. Social support from family and partners is critical for maternal and infant health and bonding [21], and emerged as a central theme. Social circles are meaningful for the emotional well-being of women in this period because social support, especially from family and partners, is a significant protective factor that can influence both the health of the mother and fetus/baby and the connection between them [21].

In this context, the multiplicity of fronts related to complexity, vulnerability, and functionality is significant, for stressful events are significant predictors of perinatal depression (PND), specifically among women who belong to ethnic groups and have low socioeconomic status [24,25,26]. For example, it was found that Bedouin women in south Israel have a higher risk of postpartum depression compared with Jewish women, due to couple conflicts and lack of support from their partners [21]. Other studies conducted in the period after the outbreak of the COVID-19 pandemic, also found high levels of vulnerability among pregnant Arab women in contrast with Jewish women on indices of emotional distress, anxiety regarding the upcoming birth, and fear of contracting COVID-19 during pregnancy [10,11].

Marginal intersectionality theory recognizes that power relationships have vital importance in building thought, experience, and knowledge, and enabling understanding and creation of different interpretations of existing facts [27,28]. The discrimination faced by the Arab sector during the war, combined with the tendency to limit women’s roles to a supportive function within marriage—whether as the supporter or the supported—highlights their marginalization. The findings indicate that, at times, women also serve as a source of support for their partners, even when they need resources. Thus, the situation of the Arab women is particularly risky, as they navigate three fronts of vulnerability, complexity, and functionality. This underscores the crucial importance of the husband’s involvement and support during such challenging times.

In addition, the theme of the impact of the war on family identity presented the increasing worry and fears that the women experienced and the emotional overload they coped with while at home during this period of war. Among the issues raised, the women indicated that they were not focused on their pregnancies but rather on other children at home, worrying about protected spaces for them in daily life situations. Some were forced to return to their families of origin to find sources of support. They also faced a fear of giving birth to their babies in a chaotic and complex situation instead of bringing them into a comfortable safe atmosphere. They coped with these challenges in the shadow of heavy economic worries and changes in their daily lives and plans.

This theme may also be explained by marginal intersectionality theory, which relates to the situation in which different axes of discrimination, such as race, ethnic background, nationality, gender, class, and sexuality, combine and interact with one another and create unique repression [29]. The different axes of discrimination include the participants’ gender identity, nationality, socio-demographic situations such as economic situation and ethno-cultural status, social processes and social systems such as policies, culture, and media [27,29]. It can be seen how, in this theme, participants found themselves in a complex reality that was unfamiliar and that they did not choose, and their being Israeli Arab women made them part of the wider Arab Muslim population that they did not necessarily fully identify with.

The fourth theme brought forward the women’s voices related to the impact of the war on socio-political identity, comprising two subthemes: the decision to be silent in view of the complex reality forced by the events and the vulnerable Arab–Jewish relationship. This theme sheds light on spaces beyond the women’s homes and neighborhoods, such as the women’s circles of work colleagues. The three identities held by the participants—being women, being Arab, and being pregnant or young mothers—led them, to a certain extent, to respond regressively, as expressed by silencing in social discourse in light of the reality they faced. Thus, the women shared that they chose to be silent and not express their positions in social conversation due to their fear of their surroundings, which was a change from how they behaved in the period before the war.

This finding aligns with previous research showing that perinatal Arab women experience improved health outcomes when supported by culturally attuned social networks. [30] emphasizes the pivotal role of social resources—including familial, spousal, and community support—in mitigating psychological distress during the perinatal period. Complementing these insights, [31] demonstrate how Bedouin women health mediators, embedded within their own communities, act as cultural and informational bridges between formal healthcare systems and traditional social structures. Together, these studies highlight the essential role of contextually grounded and culturally sensitive interventions in promoting both mental and physical health among Arab women during pregnancy and postpartum.

In addition, the existing tensions between Jews and Arabs raised a sense of fear and anxiety in participants, as they experienced a lack of understanding and stigmatizing treatment, which impacted their work functioning and negatively affected their sense of capability. These findings are significant because rates of depression have been found to be higher among women from minority groups, due to more stressful life events and lower levels of social support even in routine periods [21].

## 5. Study Limitations and Future Research

The current study has some limitations, which should be mentioned. First, the place of residence of the study participants was mainly in the north of Israel, while, at the time of the interviews, most fighting was in the south of the country. Future studies might reveal additional experiences of Arab women from other parts of Israel. Secondly, most of the participants were Muslim; therefore, expending the research to the narratives of Christian and Druze Arab women is highly recommended. Finally, most of the participants were pregnant at the time of the interviews, while some were also mothers of young children. In the future, it would be important to attend to the voices of women in different stages of motherhood.

## 6. Conclusions and Practical Implications

The themes that arose from the analysis of the interviews reveal the unique challenges faced by Arab women in Israel who are pregnant and/or in the early stages of motherhood in this conflictual complex period. They indicate that the women are coping with emotional difficulties at the personal level and challenges of couplehood and family alongside experiences of exclusion, avoidance, and silencing at the societal level. Coping with these challenges should not rest solely on the shoulders of these women but requires a combined effort on the part of health professionals, policymakers, and community organizations to ensure that pregnant women and new mothers receive the care and support they need to navigate their way through these difficult circumstances, particularly during times of war and conflict. It is crucial that health policies are adapted to address the unique needs of these women, ensuring that they have access to adequate healthcare, mental health support, and community resources during such critical periods.

A comprehensive and culturally sensitive approach to addressing the needs of Arab women during the perinatal period requires coordinated efforts across *micro*, *meso*, and *macro* levels of intervention. At the *micro* level, the critical role of individual and family support should be emphasized in mitigating stress during the perinatal period. This includes tailored stress management strategies that involve both the pregnant woman and her immediate family members, alongside family-centered educational programs. Health care providers should adopt a proactive approach by undergoing cultural competence training and offering mental health screenings and counseling that reflect an understanding of the emotional and social needs of Arab women. Furthermore, support systems such as partners and extended family should receive guidance and reinforcement, recognizing that caregivers themselves need support in order to effectively care for others.

At the *meso* level, local organizations and community-based programs can create support networks that are especially vital during times of crisis. Community-driven resilience strategies can be enhanced through the integration of psychosocial support into routine maternal health services and the deployment of mobile clinics or outreach programs for women with limited access to care. The role of cultural mediators is essential in improving communication between healthcare providers and perinatal Arab women, ensuring services are delivered with cultural sensitivity. These interventions should be embedded within the broader community context to strengthen collective responses to conflict-related challenges.

At the *macro* level, policy reforms are necessary to ensure the sustainability and effectiveness of interventions. These include increasing governmental funding for specialized perinatal services in Arab communities and expanding mental health services to include Arabic-speaking professionals. National health initiatives should raise awareness and prioritize the needs of perinatal Arab women, particularly in conflict zones. Crucially, policymakers must include Arab women’s voices in healthcare planning processes, especially in emergency preparedness and crisis response. These efforts must be guided by cultural sensitivity, addressing both the general population and the specific cultural characteristics of Arab communities.

## Figures and Tables

**Table 1:** Characteristics of study participants (*n* = 10).

No.	Age	Employment	Mother/Pregnant	Religion
1	30	Nurse	Mother to 3-month-old daughter	Muslim
2	31	Educational psychologist	Mother to 3-year-old daughter and 5 months pregnant	Druze
3	30	Human resources director	5 months pregnant	Muslim
4	28	Teacher	Mother to 7-month-old daughter	Muslim
5	33	Physiotherapist	Mother to 2 children and 9 months pregnant	Muslim
6	26	Lawyer	8 months pregnant	Muslim
7	32	Physician	7 months pregnant	Muslim
8	28	Teacher	8 months pregnant	Muslim
9	32	Nurse	Mother to 2 children and 8 months pregnant	Christian
10	25	Does not work	9 months pregnant	Muslim

## Data Availability

The datasets generated during the current study are available from the corresponding author upon reasonable request.
